# The role of magnesium in cardiac arrest

**DOI:** 10.3389/fnut.2024.1387268

**Published:** 2024-05-15

**Authors:** Baoshan Liu, Muyuan Li, Jian Wang, Fengli Zhang, Fangze Wang, Caicai Jin, Jiayi Li, Yanran Wang, Thomas Hudson Sanderson, Rui Zhang

**Affiliations:** ^1^School of Clinical Medicine, Shandong Second Medical University, Weifang People’s Hospital, Weifang, China; ^2^Department of Cardiology, Key Laboratory of Cardiopulmonary-Cerebral Resuscitation Research of Weifang, The First Affiliated Hospital of Shandong Second Medical University, Weifang People’s Hospital, Weifang, China; ^3^School of Anesthesiology, Shandong Second Medical University, Weifang, China; ^4^Department of Emergency Medicine, University of Michigan Medical School, Ann Arbor, MI, United States

**Keywords:** magnesium, cardiac arrest, resuscitation, respiratory failure, acute coronary syndrome

## Abstract

Cardiac arrest is a leading cause of death globally. Only 25.8% of in-hospital and 33.5% of out-of-hospital individuals who achieve spontaneous circulation following cardiac arrest survive to leave the hospital. Respiratory failure and acute coronary syndrome are the two most common etiologies of cardiac arrest. Effort has been made to improve the outcomes of individuals resuscitated from cardiac arrest. Magnesium is an ion that is critical to the function of all cells and organs. It is often overlooked in everyday clinical practice. At present, there have only been a small number of reviews discussing the role of magnesium in cardiac arrest. In this review, for the first time, we provide a comprehensive overview of magnesium research in cardiac arrest focusing on the effects of magnesium on the occurrence and prognosis of cardiac arrest, as well as in the two main diseases causing cardiac arrest, respiratory failure and acute coronary syndrome. The current findings support the view that magnesium disorder is associated with increased risk of cardiac arrest as well as respiratory failure and acute coronary syndrome.

## 1 Introduction

Cardiac arrest is a leading cause of death globally. In the United States, according to the American Heart Association (AHA), 146,942 individuals experience out-of-hospital cardiac arrest (OHCA) and 292,000 individuals experience in-hospital cardiac arrest (IHCA) annually (1). A recent report by the baseline investigation of out-of-hospital cardiac arrest (BASIC-OHCA) registry estimated that emergency medical service-assessed OHCAs in China have reached more than 750,000 per year (2). The two most common etiologies of cardiac arrest are respiratory failure and acute coronary syndrome (ACS). Respiratory failure accounts for the largest proportion (22%) of IHCA and the second largest proportion (12%) of OHCA. ACS and other cardiac causes account for the largest proportion (16%) of OHCA and are the third leading cause (8%) of IHCA (1, 3). Only 25.8% of IHCA and 33.5% of OHCA patients who have a return of spontaneous circulation (ROSC) following cardiac arrest survive to leave the hospital (4–6). Post-cardiac arrest syndrome is responsible for the high mortality rate among those who achieve ROSC following cardiac arrest. Post-cardiac arrest syndrome includes the following four components: (1) brain injury following cardiac arrest, (2) myocardial dysfunction following cardiac arrest, (3) systemic ischemia/reperfusion response, and (4) ongoing precipitating pathology (7, 8). There is currently no effective therapeutic approach to protect against post-cardiac arrest syndrome.

**FIGURE 1:**
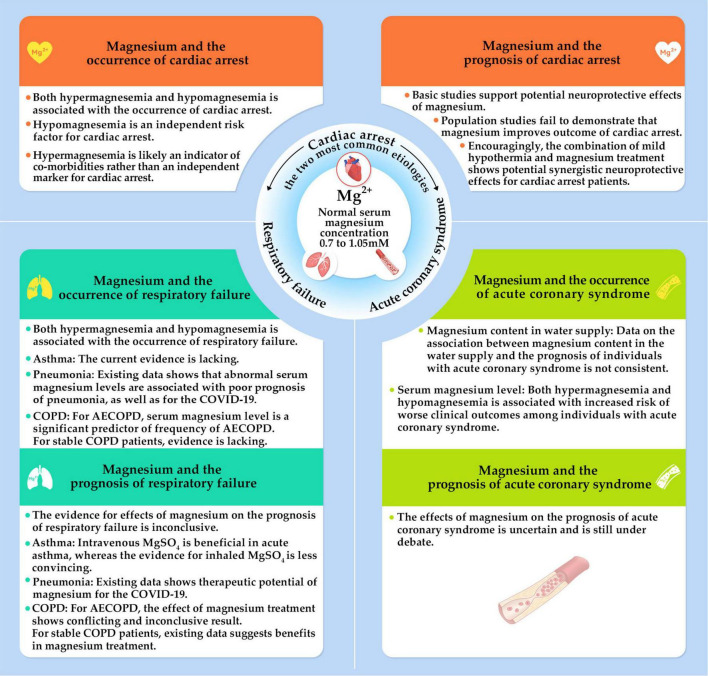
The impact of magnesium on the incidence of and prognosis after cardiac arrest. It also discusses the two most common etiologies of cardiac arrest, which are acute coronary syndrome and respiratory failure. AECOPD, acute exacerbation of chronic obstructive pulmonary disease.

**FIGURE 2:**
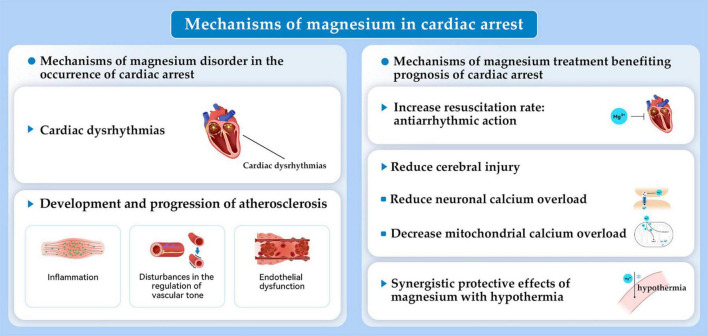
Mechanisms of magnesium in the occurrence and prognosis of cardiac arrest.

**FIGURE 3:**
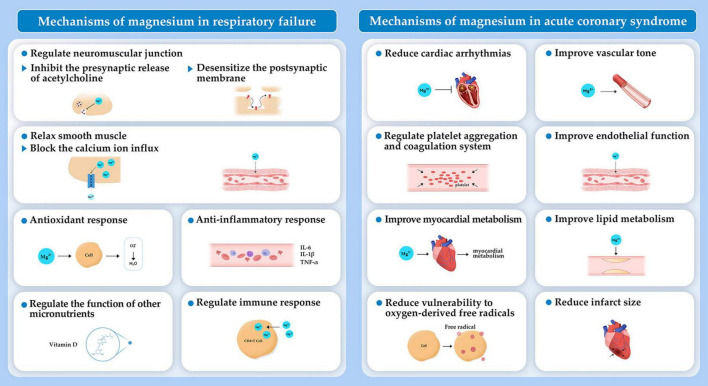
Mechanisms of magnesium in respiratory failure and acute coronary syndrome.

Magnesium is the second most abundant intracellular cation, which is essential for every organ in the human body. Magnesium participates in practically every major cellular metabolic and biochemical process including polynucleotide binding, enzymatic reactions, cell signaling, and cell proliferation (9). Research continues to examine how magnesium affects cardiac arrest. An early study found that a solution containing high concentrations of potassium and magnesium along with adenosine triphosphate, creatine phosphate, and procaine, rapidly induced arrest of isolated rat hearts and increased the resistance to periods of transient ischemia (10). Related to this, severe magnesium deficiency induced by a high-protein diet was associated with cardiac arrest in rats (11). Since then, increasing evidence has highlighted the importance of magnesium in cardiac arrest. The objectives of this comprehensive review are discussing the impact of magnesium on incidence and prognosis following cardiac arrest. Additionally, we investigate the role of magnesium in the two main diseases causing cardiac arrest, respiratory failure and ACS (Figure 1). The mechanisms by which magnesium exerts its effects are also discussed (Figures 2, 3).

## 2 Magnesium and cardiac arrest

### 2.1 Magnesium and the occurrence of cardiac arrest

Both hypermagnesemia and hypomagnesaemia are associated with cardiac arrest. Total serum magnesium concentration in healthy adults ranges from 0.7 to 1.05 mM (9). Hypermagnesemia usually occurs when creatinine clearance decreases in renal dysfunction and in instances of excessive magnesium intake from dietary supplements or magnesium containing medication. Common causes of hypomagnesaemia include a magnesium-insufficient diet, reduced intestinal absorption of magnesium, and the use of diuretics which increases the urinary excretion of magnesium (12).

Both case reports and clinical studies have discussed the relationship between levels of serum magnesium and the risk of cardiac arrest. Indeed, cardiac arrest occurred as a result of magnesium sulfate overdose for managing eclampsia (13, 14). Cardiac arrest occurred after laxative-induced hypermagnesemia in an individual with anorexia nervosa and chronic renal failure (15). Moreover, cardiac arrest was noted in an individual with emotional stress and torsade de pointes associated with hypomagnesemia (16). Clinical studies show that low serum magnesium level is a risk predictor of cardiac arrest. Results are conflicting concerning an association between hypermagnesemia and cardiac arrest. The Atherosclerosis Risk in Communities Study with subjects of 45–64 years old (*n* = 14,232) found that the risk of sudden cardiac death was higher among individuals who were in the lowest quartile (≤ 1.5 mEq/L) than those who were in the highest quartile (≥ 1.75 mEq/L) of the normal range of serum magnesium (17). Supporting these results was a prospective population-based study with a median follow-up of 8.7 years, with 9,820 participants, that showed low serum magnesium (≤ 0.80 mmol/L) was associated with an increased risk of sudden cardiac death (hazard ratio [HR]: 1.54; 95% confidence interval [CI]: 1.12–2.11; *n* = 217). The association between sudden cardiac death and high serum magnesium levels, however, was only marginally insignificant (HR: 1.35; 95% CI: 0.96–1.89) (18). The relationship between magnesium and the risk of sudden cardiac death, as measured by diet and plasma in 88,375 healthy women, was investigated in another prospective study. According to the results, higher dietary magnesium intake and plasma concentrations corresponded to reduced risks of sudden cardiac death (19). A systematic review that included eight studies encompassing 13,539 individuals with heart failure, examined the relationship between levels of serum magnesium with cardiovascular and all-cause mortality (12). An independent risk factor for sudden cardiac death is hypomagnesemia. Hypermagnesemia (serum magnesium > 2.4 mg/dl), however, was revealed an indicator for comorbidities but was not an independent marker of cardiovascular mortality (12).

The major mechanism of magnesium deficiency related with occurrence of cardiac arrest is considered to be the cardiac dysrhythmia, that magnesium deficiency alters the intracellular/extracellular K^+^ ratio resulting in a disturbance in membrane excitability due to an alteration of the resting membrane potential (20). In turn, this promotes cardiac arrest. Additionally, low levels of serum magnesium correspond to disturbances in endothelial function and vascular tone as well as to inflammation. This also may contribute to atherosclerosis development and progression, potentially causing increased heart disease mortality (Figure 2) (18). The discrepancies in the relationship between hypermagnesemia and cardiac arrest risk could be from underappreciated effects of kidney function (18). Hypermagnesemia may encourage hypotension and cardiac arrhythmias. According to recent evidence, however, increased mortality may not be attributed to hypermagnesemia as an underlying cause. It is likely to reveal comorbidities that have an impact on the prognosis of people with cardiovascular disease (12).

### 2.2 Magnesium and the prognosis of cardiac arrest

Considering the severe consequences of magnesium deficiency in cardiac arrest and the important electrophysiological functions of normal magnesium concentrations, magnesium treatment has been explored to improve the prognosis of cardiac arrest, such as successful resuscitation, short- or long-term survival, and neurological outcome. Here, we summarize studies on magnesium and the prognosis of cardiac arrest in basic studies, clinical studies, and the combined effect of magnesium and hypothermia. Current basic studies supported potential neuroprotective effects of magnesium. However, population studies failed to demonstrate that magnesium sulfate (MgSO_4_) improved cardiac arrest short- or long-term survival. Encouragingly, the combination of mild hypothermia and magnesium treatment showed potential synergistic neuroprotective effects for individuals with cardiac arrest.

In rats, MgSO_4_ reduced cerebral injury and preserved neurologic function when administered 2 days before transient global ischemia (21). Moreover, pre-treatment with MgSO_4_ was effective in resuscitation from hypoxia-induced cardiac arrest. This study showed that MgSO_4_ was beneficial because of its antiarrhythmic action during reperfusion by preventing asystole and ventricular fibrillation (22). Our group examined the single-dose intramuscular MgSO_4_ during cardiopulmonary resuscitation (CPR) in a rat model of asphyxia cardiac arrest. We explored the dose–response effect on levels of serum magnesium, neurologic function recovery, long-term survival, and neuronal loss (5). We assumed that the magnesium reduced neuronal calcium overload by directly modulating the N-methyl-daspartate receptor, preventing mitochondrial permeability transition induced by calcium, and decreasing the overload of mitochondrial calcium. A two- to fourfold increase in serum levels of magnesium was achieved within 15 min of ROSC by intramuscular MgSO_4_ during CPR. This result did not improve long-term survival, neurologic function recovery, or loss of neurons in the CA1 hippocampus. Intramuscular MgSO_4_, however, did increase the rate of 24-h survival. This result supports using intramuscular MgSO_4_ therapy during cardiac arrest and suggests that a single intramuscular MgSO4 dose may not be enough to block long-term injury pathway. In the future, whether repeated dosing strategies of MgSO4 or in combination therapy with targeted temperature management (TTM) may provide a more effective level of neuroprotection in cardiac arrest requires further investigations.

A prospective, placebo-controlled, randomized, double-blind trial of magnesium in OHCA, the MAGIC trial, found that as a first-line drug therapy, 5 g MgSO_4_ did not improve survival significantly (23). For IHCA, empirical magnesium supplementation (2 g bolus, followed by 8 g over 24 h) did not improve the resuscitation rate, 24-h survival, or survival to leaving the hospital in any subpopulation of patients with IHCA or overall (24). In particular, for prehospital individuals suffering cardiac arrest presenting with ventricular fibrillation, administration of 2 g of MgSO_4_ intravenous bolus during CPR failed to improve short- or long-term survival (25). A placebo-controlled, randomized, double-blind clinical trial featuring a factorial design evaluated whether diazepam, magnesium, or both could improve neurologic outcomes when administered immediately following resuscitation from OHCA. The results did not show a significant difference between the placebo and magnesium groups, although the people who received 2 g MgSO_4_ did experience a higher percentage of independence in daily living than the placebo controls (26). The inadequate number of individuals/group (75) was considered a factor limiting the power to detect a clinically important outcome difference. Additionally, systematic reviews did not show positive results (27–29). Therefore, there is currently no established recommendation that has be made based on these results for magnesium therapy in cardiac arrest, other than in cases in which hypomagnesemia is suspected or proven to have caused cardiac arrhythmias (30).

Targeted temperature management now has class I recommendations from the AHA for use in individuals post-ROSC. Still, significant controversies and questions remain in its implementation, including timing, target temperature, duration, method, rewarming, and side effects such as shivering, hypotension, hyperglycemia, hypokalemia, and infection (31). Based on the concept that postischemic hypothermia can have a synergistic effect to boost or unmask the neuroprotective effect of an agent (32–34), the combination effect of magnesium with TTM has been investigated in cardiac arrest. In rats, pre-treatment with magnesium before transient global ischemia was neuroprotective only in situations of mild hypothermia (35°C) (35). The use of a combination of pre- and postischemic magnesium combined with modest hypothermia (35°C) at different intervals has also been explored. Notably, postischemic treatment with magnesium and modest hypothermia (35°C) for 24 h decreased CA1 neuronal death more effectively than either treatment alone (36, 37). Clinical studies of combination therapy of magnesium with TTM in cardiac arrest are scant. Analysis of 438 survivors of cardiac arrest who completed a therapeutic hypothermia protocol, showed that lower magnesium levels at presentation and during therapeutic hypothermia were associated with favorable outcomes of cardiac arrest. Moreover, magnesium supplementation during the hospital stay was associated with improved neurological outcomes, suggesting that magnesium supplementation may potentiate the beneficial effects of therapeutic hypothermia (38). Several mechanisms explain why magnesium and hypothermia appear to act synergistically. First, both have multiple mechanisms of action following ischemia. Magnesium, for example, may maintain or increase levels of brain magnesium, thus restoring cellular parameters such as adenosine triphosphate (ATP) production, intracellular calcium, protein synthesis, and mitochondrial integrity. Meanwhile, hypothermia may restrain damage caused by free radical production, blood–brain barrier breakdown, and inflammation after ischemia (36). Furthermore, as reported in other studies which combined pharmacotherapy and hypothermia (32, 34, 39), magnesium and hypothermia therapy may prolong the therapeutic window, which supports beginning treatment several hours after cerebral ischemia. Additionally, magnesium may alleviate the side effects of hypothermia, such as reducing the shivering threshold, increasing the cooling rate, and reducing discomfort during surface cooling to 34–35°C (37, 40, 41). However, whether the combined treatment of magnesium and hypothermia could provide beneficial effects for individuals following cardiac arrest remains unclear and deserves further investigation.

## 3 Magnesium and diseases causing cardiac arrest

### 3.1 Magnesium and respiratory failure

Respiratory failure is the most common etiology of cardiac arrest (15%), which is the result of primary pulmonary pathology, including hypercarbia or hypoxia caused by exacerbation of asthma, chronic obstructive pulmonary disease (COPD), or multifocal pneumonia (3). The Global Burden of Diseases, Injuries, and Risk Factors Study (2019) found that chronic respiratory diseases, with a prevalence of 454.6 million cases globally, were responsible for 4 million deaths, making it the third leading cause of mortality worldwide (42). The five most significant lung diseases globally are asthma, COPD, lung cancer, pneumonia, and tuberculosis (43). Several treatment strategies have been studied to ameliorate symptoms, limit adverse outcomes, and increase quality of life.

Magnesium is proposed as an additive treatment for respiratory diseases because of a variety of mechanisms. Magnesium takes part in cellular homeostasis by serving as an enzymatic cofactor and involving in release of acetylcholine and histamine from mast cells and cholinergic nerve terminals. Magnesium inhibits contraction and relaxes smooth muscle by blocking calcium ion influx into the smooth muscle of respiratory system (44–46). Moreover, magnesium may regulate anti-inflammatory and antioxidant responses and assist in the proper functioning of micronutrients (e.g., vitamin D). Magnesium also plays a role in activating T cells (Figure 3). Thus, magnesium deficiency can trigger immunodeficiency, decrease antioxidant responses, exaggerate acute inflammatory response, and cause oxidative stress of respiratory system, which has been discussed widely in a recent review (47). Here, we summarize findings on the role of magnesium in respiratory failure, as well as in respiratory diseases responsible for respiratory failure, including asthma, pneumonia, and COPD in which magnesium is mostly explored.

#### 3.1.1 Magnesium and the occurrence and prognosis of respiratory failure

Magnesium is involved in the development and prognosis of respiratory failure. A significant body of research has examined the relationship between levels of serum magnesium and respiratory failure. Because magnesium can desensitize the postsynaptic membrane and inhibit the presynaptic release of acetylcholine by acting at the neuromuscular junction, it may exacerbate or cause respiratory failure and muscle weakness (48, 49). Hypermagnesemia has been responsible for prolonged respiratory failure and total flaccid paralysis when supplementing oral magnesium in the case of acute kidney injury. This result has revealed that electrolyte abnormalities may go underrecognized and lead to respiratory failure (50). When treated by magnesium replacement, a patient with hypomagnesemia and myasthenia gravis developed acute respiratory failure (48). Similarly, in another situation of myasthenia gravis and atrial fibrillation, intravenous magnesium replacement induced respiratory failure (49). Although limited, the results of these studies indicate that magnesium should be avoided or administered carefully in patients who have borderline respiratory function or myasthenia gravis.

A retrospective, single-center study examined acute respiratory failure in the case of mechanical ventilation in patients who had been hospitalized with different levels of magnesium at admission. The lowest incidence of acute respiratory failure that needed mechanical ventilation was found when levels of serum magnesium were 1.7–1.9 mg/dL. Admission hypomagnesemia (< 1.5 mg/dL) and hypermagnesemia (> 2.3 mg/dL) were both related to an increase in the risk of acute respiratory failure (51). The mechanism that hypomagnesemia causes respiratory dysfunction is that the amount of acetylcholine released from nerve terminals is decreased. Hypermagnesemia diminishes the depolarizing action of acetylcholine in the neuromuscular junction, therefore causing the bronchial smooth muscles to weaken and relax (51). The effects of different levels of magnesium on patients in the intensive care unit (ICU) with acute respiratory failure have also been studied. Neither hypomagnesemia nor normomagnesemia had a significant impact on the duration of stay in the ICU or the duration of mechanical ventilation. Statistically, however, the mortality rate of patients who had hypermagnesemia was higher than that of patients with other levels of magnesium (*P* = 0.018) (52). Given data limitations, additional research is needed to confirm these results, and cut-off values for magnesium should be further evaluated for patients who have respiratory failure.

#### 3.1.2 Magnesium and asthma

According to the latest report in 2019, asthma was the most common chronic respiratory disease with 262.4 million cases worldwide (42). To treat acute asthma, the primary therapeutic goals include reversing bronchospasm and correcting hypoxemia. Initial and conventional pharmacologic treatment of acute asthma calls for administering inhaled short-acting anticholinergic, β2-agonist and systemic corticosteroids (53). By blocking parasympathetic tone and inhibiting calcium channels, magnesium can act as a bronchodilator when administered intravenously and by inhalation. This treatment may serve as an adjunct therapy for acute asthma (53). Recent research results indicate that intravenous MgSO_4_ may improve acute asthma; however, inhaled MgSO_4_ may be less beneficial.

As an adjunct for acute asthma, intravenous magnesium has been studied with a long history (45, 54–60). To date, intravenous MgSO_4_ appears to be safe, and intravenous magnesium sulfate is considered for use in patients who have severe exacerbations (45, 54, 56, 58, 61, 62). The 2007 Cochrane review by Mohammed and Goodacre estimated the benefit of adding MgSO_4_ to β-agonists and systemic corticosteroids in children and adults who had acute asthma. They assessed 24 studies with 1,669 individuals, 15 of which included intravenous treatment and 9 included nebulized treatment (55). According to the results, intravenous MgSO_4_ treatment in adults provided only weak evidence for improving respiratory function and had no significant effect on hospital admission. Intravenous MgSO_4_ treatment in children showed reduced hospital admission and a significant improvement in respiratory function. In adults, only weak evidence was found of the effect of nebulized treatment on hospital admission or respiratory function, and in children, no significant effect was found on hospital admission or respiratory function (55). A 2008 review found that, when considering all studies, in the case of severe exacerbations, adding MgSO_4_ improved pulmonary function and reduced hospitalization (45). Another review focusing on children demonstrated that this age group was clearly benefited from intravenous MgSO_4_ (63).

For inhaled magnesium sulfate, a series of updated reviews by Rowe et al. investigated its effects for treating acute asthma from 2005 to 2017, with consistent results (44, 64, 65). The recent review including 25 trials and 2,907 randomized individuals examined whether inhaled MgSO_4_ had beneficial effects (1) when combined with ipratropium bromide and inhaled β_2_-agonist; (2) when added to inhaled β_2_-agonist; and (3) when compared with inhaled β_2_-agonist (65). However, the results did not demonstrate substantial benefits of nebulized MgSO_4_ for improved lung function or reduced hospital admission for acute asthma. Trial heterogeneity may limit the strength of the results (65). A meta-analysis of children who have acute asthma included eight randomized controlled trials (1,247 children). The findings did not suggest that inhaled magnesium offered a substantial benefit to improve lung function, limit hospital admissions, or reduce asthma symptoms or severity scores (66). A clinical trial recently published in *JAMA* also did not support any beneficial effects for children who had refractory acute asthma. When comparing nebulized magnesium with albuterol and a placebo with albuterol to treat asthma, the addition of magnesium did not decrease the hospitalization rate significantly within 24 h (67). As a result, these findings do not suggest that nebulized magnesium should be used alone or with albuterol in children who have refractory acute asthma.

#### 3.1.3 Magnesium and pneumonia

Pneumonia affects hundreds of millions of people globally. Because of the aging population, the number of hospital admissions of pneumonia has been increasing over recent decades (68, 69). The changes in serum magnesium levels in pneumonia and the impact of magnesium levels on the prognosis of pneumonia have been studied, with only limited evidence. During pneumonia, the serum magnesium level deviated markedly from the normal, while a definite loss of magnesium to the body was observed during the febrile period of pneumonia (70). Hypomagnesemia was also common in community-acquired pneumonia in individuals with type 2 diabetes mellitus. In these individuals, hypomagnesemia at admission was associated with increased short-term mortality (71). In elderly patients with community-acquired pneumonia, abnormal serum magnesium levels corresponded significantly to in-hospital mortality and 30-day mortality (68, 72). However, interventional studies are lacking to confirm the effects of magnesium treatment on pneumonia outcomes.

The coronavirus (COVID-19) pandemic, which is caused by SARS-CoV-2 infection, has brought a heavy burden to our economic and healthcare systems (73). As a result, great efforts have been made to better understand its pathogenesis and to implement low-cost prophylactic interventions. Given the multiple effects of magnesium such as anti-inflammatory and antioxidant effects, it is assumed to play a significant role in COVID-19 development and mortality (47, 73–76). Results from clinical studies found that the need for mechanical ventilation and mortality rates were higher in hypermagnesemia (magnesium levels > 2.4 mg/dL) and hypomagnesemia (<1.8 mg/dL) patients than in other patients who were hospitalized with COVID-19 (77, 78). The COMEPA study found that the low level of serum magnesium was a significant indicator of the onset of long COVID symptoms, length of stay, and in-hospital mortality (79). Levels of serum magnesium and myocardial damage were significantly negatively correlated (80). These findings indicate that measuring levels of serum magnesium in patients with COVID-19 may help predict disease-related complications. A cross-sectional study revealed that taking more dietary magnesium was inversely correlated with the severity and symptoms of COVID-19 (81). An observational study of cohorts found that combining magnesium, vitamin D, and vitamin B12 decreased the number of older patients with COVID-19 who were experiencing clinical deterioration that needed oxygen, intensive care, or both (82). Overall, current research supports the relationship between COVID-19 and imbalanced magnesium homeostasis. Therefore, robust studies are needed to investigate the therapeutic or prophylactic potential of magnesium for COVID-19 (74).

#### 3.1.4 Magnesium and chronic obstructive pulmonary disease

The primary cause of death from chronic respiratory diseases was COPD, with 212.3 million prevalent cases and 3.3 million deaths in 2019 (42). The potential clinical benefits of intravenous or nebulized MgSO_4_ for individuals with acute exacerbations or stable COPD have been studied; however, existing studies have found conflicting and inconclusive results for its effectiveness.

In patients who have acute exacerbations of COPD, serum magnesium levels were a significant predictor of the frequency of acute exacerbations, emphasizing the importance of detecting magnesium levels in individuals with COPD (83, 84). The effect of magnesium treatment on outcomes of acute COPD exacerbations has been investigated by meta-analyses. One study found that intravenous MgSO_4_ corresponded to improving dyspnea scores, reducing hospital admissions, and limiting hospital length of stay. However, no significant difference in the necessity for non-invasive ventilation was indicated, nor was a change in oxygen saturation or pulmonary function testing found (85). A Cochrane systematic review showed that magnesium infusion improved dyspnea scores and decreased the duration of hospital stay. Yet it was not clear if there was any effect on improving lung function or oxygen saturation. In contrast, magnesium inhalation, compared to placebo, did not make a difference in most outcomes of COPD exacerbations. In addition, when MgSO_4_ was compared to ipratropium bromide no differences in outcomes were noted (86). Several other systematic reviews have found either no benefit or inconclusive effects of treating acute COPD exacerbation with magnesium sulfate (87, 88). The conflicting results may be the result of the limited number of participants in these studies, as well as clinical heterogeneities in the patient populations and medication dosages and routes.

For individuals with stable COPD, several studies suggested benefits in magnesium treatment. One study showed that serum magnesium levels were associated with quality of life but not with lung function in individuals with COPD (89). Oral magnesium supplementation showed potential anti-inflammatory effects, however, this did not involve influence on lung function, physical performance, or quality of life in individuals with stable COPD (90). In patients with stable COPD, intravenous magnesium loading was correlated with decreased lung hyperinflation and improved respiratory muscle strength (91, 92). Therefore, the potential benefits of clinical magnesium supplementation for COPD warrants further study.

### 3.2 Magnesium and ACS

Acute coronary syndrome, the acute manifestation of ischemic heart disease, is a major cause of cardiac arrest (12%). In 2019, there were an estimated 673,000 ACS principal diagnostic discharges in the United States (1). Substantial progress has been made to prevent, diagnose, and treat people with ACS. Still, the burden of ACS remains unacceptably high. This situation calls for a reappraisal of the ACS related mechanisms and exploration of novel treatment therapies (1, 93). Theoretically, magnesium offers several benefits for the cardiovascular system, such as platelet aggregation and coagulation, vascular tone, endothelial function, lipid metabolism, infarct size, and cardiac arrhythmias (Figure 3), which have been discussed detailly (94–96). Here, we summarize studies on the association of magnesium with ACS prognosis from three aspects, including magnesium content in the water supply, serum magnesium levels, and magnesium administration, on the prognosis of ACS.

The relationship between magnesium content in the water supply and the prognosis of individuals with ACS, especially mortality from acute myocardial infarction (AMI), has found inconsistent results. According to data of subjects residing in England, lacked evidence of a relationship between mortality from AMI and levels of magnesium in drinking water supplies was found (97). Data from individuals in China indicated that calcium intake provided a protective effect on AMI mortality. In this case, however, no significant difference was reported in AMI mortality among the groups that had different levels of magnesium (98). In Israel, the 30-day and one-year all-cause mortality of individuals with AMI was higher in the people who were exposed to desalinated seawater, which lacks magnesium, which may reveal the contribution of reduced magnesium intake on AMI mortality (99). These people also had significantly lower levels of serum magnesium than the people who were exposed to non-desalinated drinking water. These conflicting findings could be the result of geographic differences, variance in serum magnesium levels, and confounding factors such as intake situations of magnesium supplements or medicines. Therefore, better tracking serum magnesium levels may inform the role of magnesium in ACS prognosis.

Hypermagnesemia and hypomagnesemia are correlated with an increase in worsened clinical outcomes among individuals who experience ACS. A retrospective multicenter study included more than 10,000 people with AMI and found that levels of serum magnesium were correlated with in-hospital mortality and malignant arrhythmias in a U-shaped manner (< 1.8, 1.8–1.9, 1.9–2.0, and > 2.0 mg/dL vs. 7.4, 4.1, 4.7, and 9.7%, respectively) (100). These findings show that the optimal range of serum magnesium in people with AMI may be lower than current AMI recommendations (> 2.0 mg/dL) (101). Higher serum magnesium levels at admission in individuals with reperfused AMI complicated by malignant ventricular arrhythmias were independently related with in-hospital mortality (HR: 2.68, 95% CI: 1.24–5.80). Additionally, the number of in-hospital adverse events, such as extracorporeal membrane oxygenation, cardiogenic shock necessitating intra-aortic balloon pump, persistent vegetative state or tracheostomy, and tracheal intubation, was much higher in patients with higher levels of serum magnesium than in people who had lower levels of serum magnesium (102). Since only one individual with hypomagnesemia was included, a U-shaped relationship was not observed in this study. Similarly, admission levels of serum magnesium, regardless of other risk factors, were related inversely to major adverse cardiovascular events in people who had a drug-eluting stent implantation for AMI but stable angina. Individuals with the highest levels of serum magnesium (> 0.94 mmol/L) compared with people with the lowest levels (< 0.86 mmol/L) had an 8.11-fold higher risk of major adverse cardiovascular events following implantation of a drug-eluting stent (103). A retrospective study assessed the impact of different levels of serum magnesium at admission on in-hospital mortality, including sudden cardiac death and QTc interval, in people who were admitted to the cardiac care unit with acute decompensated heart failure or a primary AMI diagnosis. A level of serum magnesium ≥ 2.4 mg/dL independently indicated an increase in hospital mortality. However, no relationship was found between levels of serum magnesium and an interval of QTc or sudden cardiac death (104). The pathophysiologic effects of hypermagnesemia and hypomagnesemia on the cardiovascular system are similar to those on cardiac arrest. However, the pathophysiologic mechanisms of magnesium remain to be addressed in individuals with ACS with different complications, including ventricular arrhythmias, drug-eluting stent implantation, and heart failure. Furthermore, some limitations need to be considered when elucidating the results: (1) the time between blood tests and administration of magnesium should be clarified; (2) measurement of serum magnesium should be uniform to allow for better comparison of results; and (3) the ability to generalize results and the statistical power to detect data differences has been limited by small sample sizes from single-center studies (102, 103).

It is still under debate whether the administration of magnesium may affect the prognosis of ACS. The Second Leicester Intravenous Magnesium Intervention Trial (LIMIT-2) was a double-blind, randomized, placebo-controlled study that included 2,316 individuals with suspected AMI and verified the benefits of magnesium (105). Notably, the administration of intravenous magnesium sulfate significantly decreased the 28-day mortality of people with AMI versus the placebo group. The Fourth International Study of Infarct Survival (ISIS-4), which was a randomized factorial trial of more than 50,000 participants, however, did not find a decrease in five-week mortality or later survival advantage among the magnesium treatment group versus the placebo group (both overall or in subgroups) (106). The proposed explanations for discrepancies between the two studies included variation in the timing and duration of treatment, the dose of magnesium, concomitant thrombolysis, and methodological problems (107). In a meta-analysis of 26 clinical trials, no advantages were found as a result of early or late magnesium treatment and outcomes (107). Although magnesium appeared to decrease the incidence of ventricular tachycardia, ventricular fibrillation, and severe arrhythmia needing treatment, it may have increased the incidence of bradycardia, flushing, and profound hypotension (107). Concerning post-ACS cardiac arrhythmias, several meta-analyses showed significant fewer reperfusion arrhythmias in the magnesium treatment groups (108–114). A recent retrospective analysis, however, revealed that magnesium did not effectively lower the incidence of reperfusion arrhythmia in people with ST-elevation myocardial infarction, who underwent primary percutaneous coronary intervention (115). One explanation for inconsistent results of this work may be the variety of revascularization techniques employed, including cardiac surgery, thrombolysis, and percutaneous coronary intervention.

## 4 Study limitations

This study has potential limitations. First, while the review covers a broad range of studies, the generalizability of these findings to different populations (e.g., different age groups, ethnic backgrounds, or geographic locations) may be limited. This could be an important limitation to acknowledge. Second, the quality of the evidence from the studies reviewed might vary. Including a critical assessment of the quality of the studies (e.g., risk of bias, methodological strengths and weaknesses) could enhance the understanding of how robust the current evidence is. Third, while the review discusses associations between magnesium levels and cardiac arrest outcomes, it is important to acknowledge that these associations might be influenced by confounding factors, such as other underlying health conditions, lifestyle factors, or concurrent treatments. Additionally, the long-term effects of magnesium therapy in cardiac arrest patients are not thoroughly discussed. The review could benefit from addressing the need for long-term follow-up studies to understand the sustained impact of magnesium. Finally, while the review outlines potential mechanisms by which magnesium impacts cardiac arrest, deeper mechanistic insights or the need for more research in this area could be a valuable addition.

## 5 Conclusion

Magnesium is an important intracellular cation that is critical for the physiological function of multiple organs including the brain, heart, and skeletal muscles. Magnesium is being tested as a treatment for cardiac arrest. This review summarized the role that magnesium can play in cardiac arrest. We assessed early research and clinical data published to date and found that a magnesium disorder indicates an increased risk of cardiac arrest as well as ACS and respiratory failure. This finding demonstrates the need for daily clinical practice to verify levels of serum magnesium. Magnesium appears to be mostly safe as an adjuvant therapy in the setting of cardiac arrest. Early results of a combination of mild hypothermia and magnesium indicate that this promising treatment may benefit individuals who are resuscitated from cardiac arrest. It continues to be controversial, however, whether or not magnesium should be used in people who are experiencing cardiac arrest as well as ACS or respiratory failure. Rigorous large cohort multi-center trials may help to clarify these issues.

## Author contributions

BL: Conceptualization, Funding acquisition, Supervision, Validation, Writing – original draft, Writing – review & editing. ML: Data curation, Validation, Writing – original draft. JW: Conceptualization, Validation, Writing – original draft. FZ: Data curation, Validation, Writing – original draft. FW: Data curation, Validation, Writing – original draft. CJ: Data curation, Validation, Writing – review & editing. JL: Data curation, Validation, Writing – review & editing. YW: Data curation, Validation, Writing – review & editing. TS: Conceptualization, Supervision, Validation, Writing – review & editing. RZ: Conceptualization, Funding acquisition, Supervision, Validation, Writing – original draft, Writing – review & editing.
